# Dengue Virus Transmission by Blood Stem Cell Donor after Travel to Sri Lanka; Germany, 2013

**DOI:** 10.3201/eid2008.140508

**Published:** 2014-08

**Authors:** Michael Punzel, Gülay Korukluoğlu, Dilek Yagci Caglayik, Dilek Menemenlioglu, Sinem Civriz Bozdag, Emre Tekgündüz, Fevzi Altuntaş, Renata de Mendonca Campos, Bernd Burde, Stephan Günther, Dennis Tappe, Daniel Cadar, Jonas Schmidt-Chanasit

**Affiliations:** MediaPark Klinik, Cologne, Germany (M. Punzel);; Public Health Institutions of Turkey, Ankara, Turkey (G. Korukluoğlu, D.Y. Caglayik, D. Menemenlioglu);; Abdurrahman Yurtarslan Ankara Oncology Hospital, Ankara (S.C. Bozdag, E. Tekgündüz, F. Altuntaş);; Federal University of Rio de Janeiro, Rio de Janeiro, Brazil (R. de Mendonca Campos); Medizinisches Versorgungszentrum Labor Dr. Quade & Kollegen, Cologne (Bernd Burde);; Bernhard Nocht Institute for Tropical Medicine, World Health Organization Collaborating Centre for Arbovirus and Haemorrhagic Fever Reference and Research, Hamburg, Germany (S. Günther, D. Tappe, D. Cadar, J. Schmidt-Chanasit);; German Centre for Infection Research, Hamburg-Luebeck-Borstel, Hamburg (J. Schmidt-Chanasit)

**Keywords:** Dengue, viruses, RNA, *Flaviviridae*, arthropod, mosquito, vectorborne, blood, stem cell, donor, transplant, fatal, PCR, Germany, Sri Lanka

## Abstract

Three days after donation of peripheral blood stem cells to a recipient with acute myeloblastic leukemia, dengue virus was detected in the donor, who had recently traveled to Sri Lanka. Transmission to the recipient, who died 9 days after transplant, was confirmed.

Dengue virus (DENV), an arthropod-borne RNA virus of the *Flaviviridae* family, has 4 serotypes that cause dengue fever or dengue hemorrhagic fever in humans. DENV has become a worldwide public health problem: current estimates indicate 390 million DENV infections and 96 million clinically apparent cases in 2010 (*1*). The virus is found in tropical and subtropical regions around the world and is hyperendemic to areas in Asia and Latin America (*1*). 

Hematopoietic stem cell transplantation has become a major treatment option for patients with hematopoietic malignancies and immune deficiencies. Each year, >50,000 allogeneic transplants are performed worldwide (*2*). Despite mandatory testing of donors and strict exclusion criteria to prevent transmission, risk remains for transmission of communicable diseases, including tropical diseases for which screening is not usually performed. To the best of our knowledge, only the transmission of malarial parasites has been reported during stem cell transplantation (*3*,*4*). Here, we report transmission of DENV to a peripheral blood stem cell recipient by a donor who had recently traveled to an area to which the virus is endemic. We recommend testing of recent travelers returning from areas to which DENV is endemic before allowing such donations.

## The Study

Acute myeloblastic leukemia was diagnosed in a 51-year-old man in Germany in September 2012. According to international standards, cytogenetic and molecular examination determined that this form of leukemia was “poor risk” at the time of diagnosis. Because of the patient’s risk status, the physicians intended to perform allogeneic stem cell transplantation after induction and consolidation chemotherapy, which was scheduled to end in January 2013, and a conditioning chemotherapy regimen, which was planned to be given in March. Because of lack of a related HLA-matched donor, an international donor search was performed; 1 fully matched unrelated female donor was identified in the German National Registry. The 24-year-old woman, who was registered as a volunteer donor in the German Bone Marrow Donor Registry, was selected.

The donor had scheduled a trip to Sri Lanka, and was to return 3 days before the scheduled start of granulocyte-colony-stimulating factor (G-CSF) application. According to German (http://www.zrkd.de/de/_pdf/ZRKD_Standards-V9_deutsch.pdf) and international (http://www.worldmarrow.org/fileadmin/Committees/STDC/20140101-STDC-WMDA_Standards.pdf) guidelines, such travel should have led to the postponement of donation because many infectious diseases are endemic to Sri Lanka. However, the donor was unable to postpone her trip, and the recipient was in urgent need of the transplant. The transplant physicians agreed to keep the dates as scheduled and confirmed the exception as Declaration of Urgent Medical Need of the transplant.

Five days before the scheduled transplant (day –5), the recipient tested positive for *Klebsiella pneumoniae* infection of the central venous catheter. The catheter was removed and piperacillin/tazobactam treatment was initiated. 

The donor had returned from her trip 3 days before the start of G-CSF-injections without any signs of infection. On the day of apheresis (day 0), the donor showed signs of a respiratory infection with axillary temperature of <39°C, bone pain, and headache. Oral azithromycin (2 × 250 mg/d) and ibuprofen 400 mg were given. The stem cell mobilization result was poor: 11 CD34+ cells/μL peripheral blood (0.04% of leukocytes) were measured at the beginning of apheresis. Before apheresis, the donor’s blood count showed mild thrombocytopenia (134,000 cells/μL) after G-CSF mobilization. Standard leukopheresis processing of 14 L of blood from the donor was performed without problems. Approximately 90 × 10^6^ CD34+ cells, corresponding to 10^6^ CD34+ progenitor cells per kg bodyweight of the recipient, could be collected. 

A second apheresis or a bone marrow collection was considered, but neither was performed because the clinical condition of the donor worsened. Her temperature increased to 41°C, the platelet count dropped from 84,000 cells/μL on day 0 to 74,000/μL the day after. In the morning of the second day after apheresis (+2), the platelet count dropped to 47,000/μL, procalcitonine level was elevated at 1.10 μg/L, C-reactive protein level was elevated at 10.5 mg/L, and a slight skin rash developed. Because of the clinical course, on day +1, physicians suspected a possible DENV infection. A serum sample test showed a weak positive result for DENV by using IgM and IgG antibody tests (in-house indirect immunofluorescence assay), as used by Tappe et al. (*5*), and a strong positive result for DENV nonstructural protein-1 (NS1) antigen, demonstrating acute DENV infection. Quantitative real-time reverse transcription PCR for DENV RNA (*6*) was positive and showed a DENV RNA load of 2.6 × 10^10^ copies/mL. Testing of the sample containing the progenitor cells showed a DENV RNA load of 4.8 × 10^8^ copies/mL.

After being informed about possible infection of the donor, the transplant physicians administered immunoglobulin to the recipient intravenously (0.5 g/kg/y for 4 days). At posttransplantation day +3, antibiotic drug therapy was switched from piperacillin/tazobactam to meropenem. On the same day, physical examination revealed painful hepatomegaly and increased total bilirubin, diagnosed as hepatic veno-occlusive disease (Table). Therefore, defibrotide prophylaxis, which had been initiated on day –8, was increased to treatment dose. On day +7, empiric antifungal therapy was added. On the same day, *Staphylococcus epidermidis* was detected in blood cultures and vancomycin was given. On day +8, the recipient experienced severe abdominal pain accompanied by hematochezia, hypoxia, and metabolic acidosis. Bacteriologic culture of a tracheal aspirate grew *Acinetobacter baumannii,* which was only susceptible to colomycin and tigecyclin. The recipient was transferred to the intensive care unit and died from cardiopulmonary arrest 9 days posttransplant. A blood sample from the recipient on day +3 was retrospectively analyzed and tested negative for DENV IgM and IgG but positive for DENV NS1 antigen and DENV RNA with a DENV RNA load of 8.6 ×10^7^ copies/mL. Sequencing of the DENV amplicons from all samples demonstrated a DENV serotype 1 (DENV 1) genotype 1 infection of the donor and recipient. Phylogenetic analysis of the complete envelope protein coding gene of DENV 1 strains revealed that the DENV 1 genotype 1 strains detected in the donor were closely related to currently circulating DENV 1 genotype 1 strains in Sri Lanka (Figure).

**Table:** Timeline of events before and after allogeneic stem cell transplantation for patient and donor, Germany*

Time before (–.) and after (+) transplantation	Recipient event	Donor event
Month ­­–5	Diagnosis of poor risk acute myeloblastic leukemia and introduction of chemotherapy	Related donor assessment
Months –5 to –2	Consolidation chemotherapy	Unrelated donor search
Month –2	NA	Request of confirmatory typing (CT) and workup of the donor
Day –28	NA	Medical examination without any abnormalities
Day –25 to day –9	NA	Traveled to Sri Lanka
Day –8	Conditioning regimen started (defibrotide prophylaxis)	NA
Day –5	*Klebsiella pneumoniae* infection of the central venous catheter; catheter was removed and piperacillin/tazobactam was given	NA
Day -6 to day –2	NA	G-CSF mobilization of the donor
Day –1	NA	Stem cell collection; fever of unknown origin developed
Day 0	Transplantation of the stem cell graft; information about fever of the donor	Donor condition worsened suspect of tropical disease with thombocytopenia, skin rash and fever <41°C
Day +3	Fever and clinical signs of hepatic veno-occlusive disease with subsequent treatment	DENV infection confirmed by laboratory
Day +7	*Staphylococcus epidermidis* in blood cultures with subsequent vancomycin treatment	NA
Day +8	Hematochezia, metabolic acidosis, hypoxia, abdominal pain, and intensive care unit	Donor slowly recovered from DENV infection
Day +9	Patient died from enterocolitis/hepatic veno-occlusive disease	NA

*NA, not applicable.

**Figure:**
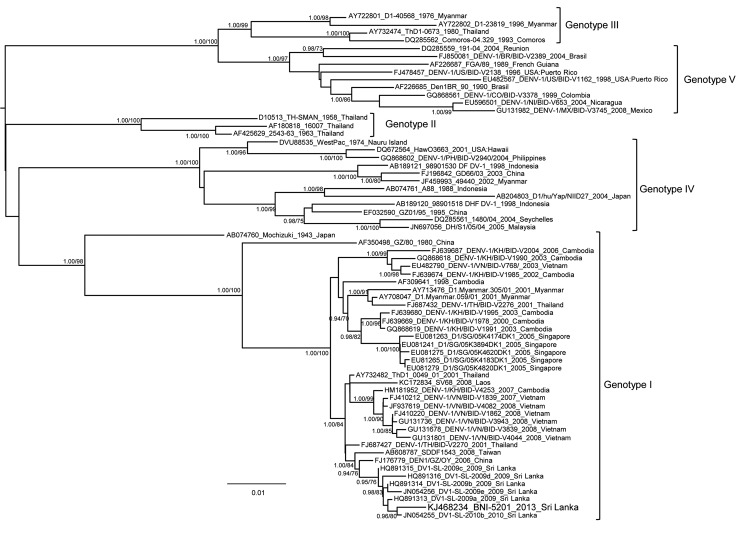
Bayesian phylogenetic tree based on complete envelope protein coding gene of dengue virus 1 (DENV-1) serotype. The tree was constructed by using the Bayesian Markov Chain Monte Carlo sampling method and BEAST software (http://beast.bio.ed.ac.uk). The general time reversible model of sequence evolution with gamma-distributed rate variation among sites and a proportion of invariable sites and a relaxed (uncorrelated log-normal) molecular clock model were used. Bayesian posterior probabilities and percentages of replicate trees in which the associated taxa clustered in the bootstrap test (1,000 replicates) obtained by using a maximum-likelihood analysis are shown at the branches. Strains are denoted by GenBank accession number, name, year of isolation, and country of origin. The strain BNI-5201 from this study is highlighted. Scale bar indicates mean number of nucleotide substitutions per site.

## Conclusions

This case demonstrates the transmission of DENV by allogeneic blood stem cell transplantation. However, although the transmission of DENV was demonstrated, the patient’s death was probably caused by hepatic veno-occlusive disease and toxic enterocolitis related to the conditioning regimen. 

To avoid transmission of tropical viruses such as DENV, under German Federal Ministry of Health rules, blood and stem cell donors are excluded from donation 4 weeks after returning from areas to which such disease agents are endemic (*7*). DENV has an incubation period of 3–14 days, and the risk for transmission of such viruses under this exclusion is very low. Few cases of DENV transmission by blood transfusion or organ transplantation have been published or reported (*8*–*11*). This case represents a difficult situation: a patient in urgent need of a lifesaving transplant that must be performed without delay, and the only matched donor scheduled for travel to a region to which DENV is endemic. The physician decided to proceed with the scheduled transplantation date because of the urgent need of his patient, although he was aware of the risk for transmission of tropical diseases. 

In such situations it is difficult to estimate the risk/benefit ratio, so it will require a case-by-case decision between donor interests and recipient needs. All diagnostic tools should be used to minimize the risk for viral transmission before transplantation. This could have been easily accomplished in this case, because preprocedure samples from the donor tested positive for DENV NS1 antigen. Thus, we recommend highly sensitive and specific testing for DENV NS1 antigen (*12*) of every donor returning from regions to which DENV is endemic for DENV NS1 antigen if transplantation cannot be postponed because of urgent medical need.
